# *In Vitro*/*In Vivo* Toxicity Evaluation and Quantification of Iron Oxide Nanoparticles

**DOI:** 10.3390/ijms161024417

**Published:** 2015-10-15

**Authors:** Ujwal S. Patil, Shiva Adireddy, Ashvin Jaiswal, Sree Mandava, Benjamin R. Lee, Douglas B. Chrisey

**Affiliations:** 1 Department of Chemistry, University of New Orleans, 2000 Lakeshore Drive, New Orleans, LA 70148, USA; 2 Department of Physics and Engineering Physics, Tulane University, 5050 Percival Stern Hall, New Orleans, LA 70118, USA; E-Mails: sadiredd@tulane.edu (S.A.); douglasbchrisey@gmail.com (D.B.C.); 3 Department of Immunology, the University of Texas MD Anderson Cancer Center, 7455 Fannin Street, Houston, TX 77054, USA; E-Mail: arjaiswal@mdanderson.org; 4 Department of Urology, Tulane University School of Medicine, 1430 Tulane avenue, SL-42, New Orleans, LA 70112, USA; E-Mails: smandava@tulane.edu (S.M.); brlee@tulane.edu (B.R.L.)

**Keywords:** toxicity, quantification, iron oxide nanoparticles, physicochemical properties

## Abstract

Increasing biomedical applications of iron oxide nanoparticles (IONPs) in academic and commercial settings have alarmed the scientific community about the safety and assessment of toxicity profiles of IONPs. The great amount of diversity found in the cytotoxic measurements of IONPs points toward the necessity of careful characterization and quantification of IONPs. The present document discusses the major developments related to *in vitro* and *in vivo* toxicity assessment of IONPs and its relationship with the physicochemical parameters of IONPs. Major discussion is included on the current spectrophotometric and imaging based techniques used for quantifying, and studying the clearance and biodistribution of IONPs. Several invasive and non-invasive quantification techniques along with the pitfalls are discussed in detail. Finally, critical guidelines are provided to optimize the design of IONPs to minimize the toxicity.

## 1. Introduction

The commercial use of engineered nanoparticles (NPs) has increased extensively in recent years to include applications in areas such as advanced nanoelectronics, optics, enhanced automation and robotics, nanostructured chemical catalysts, textile, oil and gas industries [1,2]. Moreover, nanotechnology have seen tremendous advancements and proven to be a potential interest for pharmaceuticals applications including drug delivery and drug developments [3,4]. Of all types of NPs, magnetic nanoparticles (MNPs, 100 nm or less in diameter) have been widely accepted in the biomedical field due to its tunable size and shape, superparamagnetic nature, simple synthesis, high surface to volume ratio, and ability to effectively carry a broad range of biological ligands [5].

Superparamagnetic iron oxide MNPs (magnetite, Fe_3_O_4_ and maghemite, γ-Fe_2_O_3_) have been used in numerous applications in biomedicine, such as magnetic resonance imaging (MRI) [6], drug delivery [7], and thermoablation therapy [8]. IONPs can be coated with hydrophilic layers followed by subsequent conjugation of target specific proteins (peptides, antigens and antibodies), which add much needed biocompatibility and target specificity [9]. By conjugating certain vectors such as target specific peptides or using magnetic field, IONPs can cross the blood brain barrier (BBB) and can be utilized for drug delivery [10] or diagnosis of Alzheimer’s disease by molecular imaging [11]. Superparamagnetic IONPs have also been used in various biosensing applications to target a broad range of bacteria, viruses, nucleotides, proteins and cancer cells [12]. Moreover, IONPs have been widely utilized in enrichment and sample preparation steps in proteomics [13,14] and genomics [15].

As the development of IONPs in biomedicine and manufacturing industries continue to rise, so do the risks of environmental damage and biological toxicity as these particles accumulate in the environment, the food chain, and the work force [16]. Indeed, many IONPs are attractive therapeutic anti-cancer agents precisely because they cannot be metabolized and are ultimately cytotoxic; the very properties that are technologically important are also toxic *in vivo*. Yet, the off-target impact of IONPs exposure is largely unknown because the mechanisms of IONPs interactions with otherwise healthy cells, tissues, and organisms are not well understood [17]. Because of the potential damage that engineered NPs can cause to the environment and personal health, it is critical to monitor the environment for potential threats.

Considering the potential and growing applications of MNPs, many government and non-government agencies have introduced regulatory policies facilitating the scientific advancement and promoting safe use of nanotechnology based medicine. A plethora of information is being generated to explain the impact of physiochemical properties of IONPs on toxicity and bio-distribution pattern of IONPs. The Nanotechnology Characterization Laboratory (NCL), which is a combined effort by FDA, National Cancer Institute (NCI), and National Institute of Standards and Technology (NIST) has developed a standardized analytical cascade that include physical *in vitro* and *in vivo* characterization of NPs. Specifically, NCL carry out pre-clinical characterization of nanomaterials intended to diagnose or cure cancer [18] In another worthy attempt, the European commission initiated the NanoTEST project in 2008, which focused on studying the interaction of NPs, including IONPs, and developing the high throughput tools to study nanotoxicity using *in vitro* tools. The need to evaluate the biotransformation of IONPs in the body and their pharmacokinetic profiles was strongly emphasized in the NanoTEST project [19]. Many unanswered questions regarding the toxicity of IONPs can be answered by a deeper understanding of the role of physicochemical properties of IONPs in their interaction with living cells and their distribution pattern in the body.

The current review aims to explain the correlation between the mechanism of toxicity of IONPs and major physicochemical factors responsible for *in vitro*/*in vivo* toxicity. Considering the significant impact of physicochemical parameters on the absorption, distribution, metabolism and excretion (ADME) pattern of IONPs, it is required to quantify each unique IONP containing nanomedicine. Thus, this document also discusses a variety of invasive and noninvasive quantification techniques along with their detection limits and will assist readers and scientists to choose an appropriate technique for *in vitro*/*in vivo* quantification of bare and coated IONPs.

## 2. Mechanism of Toxicity of Iron Oxide Nanoparticles (IONPs)

Findings from the majority of nanotoxicity studies conducted with IONPs have found the production of reactive oxygen species (ROS) as a major reason behind cell death. A variety of stress factors such as temperature, interaction with pathogens, and foreign materials can be responsible for the generation of ROS. ROS such as anions, hydroxyl radicals, and hydrogen peroxide are byproducts of oxidative metabolism, which occurs in mitochondria. Cells respond to increased levels of ROS by a “detoxification” mechanism, which involves enzymes such as superoxide dismutase and glutathione peroxidase. Glutathione has been considered to play a major role in defense against ROS [20]. It oxidizes upon interaction with ROS and the reduced form is regenerated by NADPH dependent reductase. Thus, the ratio of oxidized and reduced form of glutathione can be used as an indicator of overproduction of ROS. Overproduction of ROS has been linked to lipid peroxidation [21], DNA strand breaks [22], alteration in gene transcription [23], and generation of protein radicals [24]. Excessive ROS is known to affect the immune system and has been linked with various illnesses including cardiovascular diseases [25], cancer [26], inflammatory illnesses [27], diabetes [28], Parkinson's disease [29], and arthritis [30]. Several research studies aiming to investigate the IONPs-induced ROS production proposed a few mechanisms such as peroxidase-like activity of IONPs promoted in acidic environment of lysosomes, interaction of iron ions with mitochondria, and activation of cell signaling [31].

Systematic administration of IONPs in the blood stream faces the initial uptake by the liver and spleen. IONPs uptake is mediated by the mononuclear phagocytic system via endocytosis in kupffer cells of the liver and macrophages of spleen. IONPs are degraded in the lysosomes of kupffer cells and macrophages, thereby releasing the free iron from IONPs, which affect the iron homeostasis. IONPs can be enzymatically degraded in the lysosomes (pH~4.5) and released iron ions can participate in the Fenton reaction to produce hydroxyl radicals [32,33]. Free iron is stored in the form of proteins, such as ferritin and haemosiderin, for further use in the body. However, when the iron storage capacity of these proteins is exceeded, the body experiences iron overload which mainly triggers the production of the ROS [34].

### 2.1. Shape, Size and Surface Chemistry of IONPs

Physicochemical parameters of IONPs such as size, shape, and surface chemistry also contribute towards ROS induction in cells. The higher surface area of smaller IONPs has been linked to increased toxicity of IONPs [35,36]. However, some studies have found no significant difference in the size-dependent toxicity of IONPs. The bare Fe_3_O_4_ (20–30 nm, surface area: 42 m^2^/g) and Fe_3_O_4_ (5 μm, surface area: 6.8 m^2^/g) have toxicity in A549 cells in terms of cell death, mitochondrial damage, and DNA damage. But, no significant difference was found between the toxicity response by Fe_3_O_4_ (20–30 nm) and Fe_3_O_4_ (5 μm) [37]. The shape of IONPs also has a varying degree of response towards toxicity as rod shaped IONPs (Fe_2_O_3_) showed a higher degree of necrosis in mouse macrophage cells than spherical IONPs did. Rod-shaped IONPs were mostly accumulated in the cytoplasm, while spherical IONPs aggregated in vacuoles. Higher surface area/volume, nonspecific endocytosis, and membrane damage due to their rod shape can explain the higher toxicity compared to spherical shaped IONP [38]. The surface chemistry of bare and coated IONPs has also been considered to be an important factor that affects the toxicity of IONPs [33]. The direct interaction of bare IONPs could also be responsible for leaching of more iron, resulting in iron overload. Numerous studies have appeared in literature to differentiate between the toxicity of bare and coated IONPs. These studies involved IONPs synthesized by different techniques, coating agents, types of tissues/cells, and cytotoxicity assays.

Due to this diversity, it has been extremely difficult to assign a definite toxicity profile that can be followed while choosing the proper IONPs. Several reports discussed the higher toxicity of bare IONPs than coated IONPs; however, some research studies have found the toxicity of bare IONPs to be less than that of the (oleate) coated IONPs. Oleate itself did not show any cytotoxicity, which shows that the toxicity might have been associated with the interaction and cellular uptake of oleate coated IONPs [39]. Incorporating a layer on bare IONPs can certainly reduce the toxicity levels by reducing the oxidative stress and alterations in iron homeostasis [40], but the direct role of surface passivation of IONPs has not been clearly understood. Some researchers speculated that the extra layer reduced leaching of iron ions and the lysosomal degradation of iron ions [41].

### 2.2. Chemical Nature of IONPs

The chemical composition and crystalline nature of IONPs have also mediated ROS linked redox reactions. The Fenton-like reaction was significantly affected in terms of increased H_2_O_2_ production by the higher ratios of iron (II, III) at neutral pH levels [42]. Moreover, the stoichiometric ratio of Fe^2+^ and Fe^3+^ [43] and oxidation states (magnetite and maghemite) respond differently toward the redox activity and production of hydroxyl radicals [33,44]. Recent work performed with alveolar macrophage cells showed increased ROS, nitric oxide, and cytokine production upon IONPs exposure and also resulted in mitochondrial and morphological damage by magnetite IONPs compared to maghemite IONPs. In the face-centered cubic structure of magnetite, Fe^3+^ occupies all the tetrahedral sites, and Fe^3+^ or Fe^2+^ occupies the octahedral sites. However, in maghemite all the octahedral sites have cationic vacancies and mostly contain Fe^3+^. Thus, dissolution of magnetite IONPs releases Fe^3+^ or Fe^2+^ while maghemite releases Fe^3+^ only [45]. Different crystalline phases such as Fe_2_O_3_ and α-Fe_2_O_3_ have shown a varying degree of hydrogen peroxide degradation, later being more catalytic [46].

### 2.3. Morphological Changes Induced by IONPs Exposure

Exposure of IONPs can cause alterations in cell morphology, cytoskeleton of cells, and cell motility [47,48]. Endothelial cells were elongated to twice their original length when reacted with IONPs for 12 h [49]. Zhang *et al*. suggested that the adsorption of IONPs on cell surfaces can be responsible for disturbing the structure and function of the cell membrane. Size and time dependent disruption of junctional complexes in the epithelium of caco-2 cell lines was verified by transepithelial electrical resistance measurements (TEER). The disruption was found to be maximum with high doses (300 mg/L) and 26 nm IONPs, and the γ-catenin, which is a key protein in adherence junctions, was also affected [50]. The same group of researchers further observed a higher degree of disruption of endothelial integrity (fallen microvilli and clumping of microvilli) in 17 nm IONPs than in 53 nm IONPs [51]. Astanina *et al.* described the impaired cell integrity of HMEC-1 and HUVEC cells upon incubation with IONPs. Decreased impendence and increased intracellular gap junction was observed, which is an indication of altered cytoskeleton and plasma membrane, and impaired endothelial barrier [52].

### 2.4. Genotoxic Effects of IONPs

Genotoxic effects of IONPs are mainly influenced by direct interaction with leached iron ions from IONPs or various indirect factors, such as excessive ROS [53] and IONPs induced cellular stress. Direct and indirect contact of IONPs with DNA can affect the structure of DNA (strand breaks, crosslinking, and oxidation of nucleotides) as well as DNA transcription and replication. In addition to DNA damage, IONPs upregulate genes that are associated with endothelial layer integrity [51] and lysosomal function [54], activated caspase [55,56] and cytokines [57], and changes in iron metabolism related genes [58]. Physicochemical properties of IONPs such as size [59,60], surface chemistry [39,61,62], chemical composition [63], crystalline nature, and dispersity of IONPs can significantly affect the genotoxicity profile of IONPs. Various types of genotoxicity assay protocol and types of cells and mediums can also have an impact on the outcome.

### 2.5. Role of Protein Corona on ROS Formation

Even though the physicochemical properties of IONPs have a major influence on the toxicity of IONPs, the role of cell-nanoparticle interactions and protein corona has also affected the *in vivo* toxicity of IONPs. Once IONPs come in contact with blood, various proteins are adsorbed on the surface of IONPs, forming a protein corona. Adsorption of proteins can be affected by size, charge [64], source of protein adsorbed [65], incubation temperature [66] and type of cell medium on the IONPs [67]. Studies conducted with human alveolar epithelial cells (A549) showed the role of surface groups on IONPs in protein corona formation and its dose dependent effect on oxidative stress. Authors claimed that the bare IONPs incubated in fetal bovine serum (FBS) produced less amount of oxidative stress as compared to the bare IONPs incubated in synthetic serum. This can be attributed to the protein corona formed on the surface of IONPs during incubation with FBS [68]. However, more work need to be performed in this field to establish a clear relationship between the protein corona formation and surface functional groups of IONPs.

Recently, a group of researchers studied the effect of slight temperature changes on the protein corona formation of IONPs. Protein coronas formed at different temperature showed different responses towards ROS generation in HeLa cells. Positively charged IONPs generated more ROS compared to neutral and negatively charged IONPs, highest being at 39 °C. These results were further supported by analysis of neutral, negatively and positively charged IONPs in HeLa cells using LysoTracker assay and electron microscopic analysis [69]. The response of protein corona towards ROS generation and cell death is complex. Even though the role of protein corona in reducing toxicity has been demonstrated earlier, it is difficult to definitively point out the mechanism due to a variety of physicochemical factors involved in this complex interaction.

Undoubtedly, the catalytic properties of iron play a major role in the induction of ROS formations resulting into oxidative stress. Passivating the surface of IONPs can certainly alter the ROS production but, is not the only factor to control it. In addition to physiochemical properties of IONPs, various experimental conditions also influence the production of ROS and require careful monitoring to narrow down the responsible factor.

## 3. *In Vitro* and *in Vivo* Techniques to Evaluate Toxicity of IONPs

Toxicity studies of IONPs started to appear during late 1980s when IONPs were introduced as MRI contrast agents [70,71]. Initially, IONPs were considered biocompatible and non-toxic due to their resemblance to endogenous iron in the body. This was due to the fact that the contribution of physicochemical properties of IONPs towards the biocompatibility and toxicity of IONPs was untouched since the focus of the majority of studies was on understanding the mechanism of cellular uptake of IONPs. Focus of the IONPs research continued on synthesizing a variety of nanostructures with diverse surface chemistries to facilitate the conjugation of biomolecules to the surface of IONPs. In the last two decades, IONPs have seen momentous developments in terms of various shapes, sizes, coating layers, and functional groups. Variety of organic polymers containing coating agents has been introduced to produce hydrophilic NPs, which are more suitable for clinical applications. To improve biocompatibility and reduce cytotoxicity, the surface energy and reactivity of IONPs was manipulated [72]. Apart from these efforts, a little success was achieved when only a few superparamagnetic IONPs have been FDA approved for clinical use [73,74]. Translation to other disciplines is limited by the disadvantages exhibited by concentration dependent toxicity [75,76]. Tremendous variations in the physicochemical properties of IONPs have affected the cell-NPs interactions, thereby affecting the toxicity profiles. It is critical to understand the potential risks involved with the interaction of IONPs. Regardless of the disadvantages, potential exists, and *in vivo* research is currently underway to explore the feasibility of using IONPs in a delivery and ablative treatment capacity. Magnetic localized delivery of drug-loaded NPs for cancer therapy is advantageous due to the nanoparticle’s design in bypassing cellular uptake barriers. Efficient treatment utilizing magnetic ablation has been shown in xenograft models [77].

### 3.1. In Vitro Toxicity Evaluation of IONPs

Researchers aim to solve the puzzle of nanotoxicity by collecting the data obtained from *in vitro*, *in vivo*, and clinical nanotoxicological studies. Even though *in vitro* studies do not substitute for *in vivo* or clinical studies, it is considered a good starting point for toxicity studies. Currently, toxicity studies are considered a mandatory factor while demonstrating the use of IONPs intended to diagnose or treat illnesses in the human body. Primary *in vitro* studies conducted with IONPs shed light on changes in membrane integrity, metabolic activity, and genetic material of cells upon reacting with IONPs. *In vitro* nanotoxicity assessments can produce reliable and reproducible results without the use of animals, and are of simple, rapid, and inexpensive nature. The (3-(4,5-dimethylthiazol-2-yl)-2,5-diphenyltetrazolium bromide) (MTT) or (3-(4,5-dimethylthiazol-2-yl)-5-(3-carboxymethoxyphenyl)-2-(4-sulfophenyl)-2*H*-tetrazolium) (MTS) assay, which measures the mitochondrial function, and lactate dehydrogenase (LDH) assay, which measures the cell membrane integrity, are widely used cell-based assays in studying the toxicity of IONPs. Reproducibility is highly affected by certain parameters such as types of NPs, cells, cell culture conditions, and assay protocols. Results of *in vitro* assays can also be affected by the large surface area and chemical nature of IONPs, which can interact with MTS [78], affect the Cl-ion in cell culture medium [79], and interfere with colorimetric absorbance based measurements [80,81].

**Figure 1:**
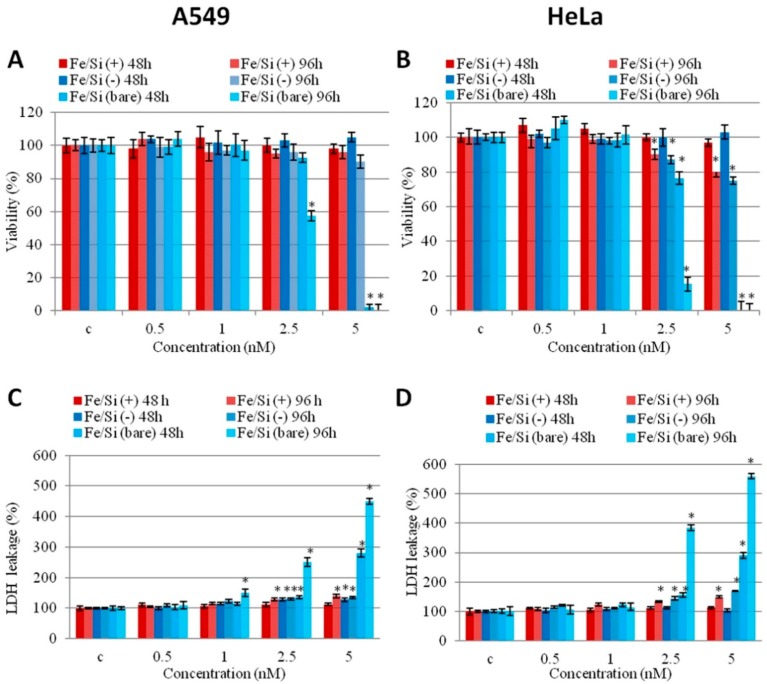
Effect of bare and passivated Fe_3_O_4_/SiO_2_ nanoparticles (NPs) on the viability and membrane damage in two cell lines (A549 and HeLa). (**A**,**B**) (2-(2-Methoxy-4-nitrophenyl)-3-(4-nitrophenyl)-5-(2,4-disulfophenyl)-2*H*-tetrazolium (WST-8) proliferation assay and (**C**,**D**) Lactate dehydrogenase (LDH) assay on A549 and HeLa cells incubated with increasing concentrations (0.5, 1, 2.5, 5 nM) of bare and passivated Fe_3_O_4_/SiO_2_ NPs at different times (48 and 96 h). c identifies the negative control in the absence of NPs. Viability of NPs-treated cells is expressed relative to non-treated control cells. As positive control (P) cells were incubated with 5% dimethyl sulfoxide (DMSO) in WST-8 assay and 0.9% Triton X-100 in LDH assay (not shown). Data are reported as mean ± SD from three independent experiments; * *p* < 0.05 compared with control (*n* = 8). Reprinted with permission from Malvindi *et al*. [40]. Copyright 2015 PLoS One-Public library of science.

Present toxicological information reflects the importance of meticulous physicochemical characterization of IONPs and its effect on *in vitro* toxicity studies. The size, shape, charge, surface area, and aggregation of IONPs have a significant impact on *in vitro* toxicity studies. Increased toxicity was observed with polyethylimine coated IONPs (50 μg/mL), whereas inclusion of PEGylation and acetylation eliminated cytotoxicity in KB cells (MTT assay). Authors claimed that the increase in toxicity can be attributed to the strong electrostatic interaction between the negatively charged cell surface and positively charged IONPs at higher doses [82]. Numerous studies have discussed the increased cytotoxicity of surface-passivated IONPs over bare IONPs [83,84,85]. Dextran, silica, and PEG have also shown reduced toxicity in *in vitro* tests. Figure 1 shows the cell viability after treatment with bare and silica-coated IONPs. Bare IONPs reduced the cell viability (A549 and HeLa cells) at higher concentrations, whereas silica-coated IONPs did not induce any cytotoxicity. Even though the surface coatings of IONPs have shown reduction in cytotoxicity, this response changes according to the size of IONPs. The bare IONPs (30 nm, 0.5 mg/mL) induced higher ROS formation compared to bare IONPs (5 nm, 0.5 mg/mL) in porcine aortic endothelial cells (PAEC), whereas dextran and PEG coated IONPs did not show any changes in ROS at similar concentrations. The same study also reported cell elongation and actin cytoskeleton disruption upon exposure to bare IONPs (30 nm, 0.5 mg/mL) [83].

Furthermore, the type of functional groups adds another complication to the *in vitro* test results. Due to the widespread use of positively charged, amine modified IONPs and negatively charged, carboxyl group modified IONPs in biomedical applications, majority of toxicity studies were performed with these types of IONPs. Amine-modified IONPs have been found to be more lethal in *in vitro* tests due to their strong interaction with negatively charged cell surface [86,87]. However, COOH modified IONPs have also shown some toxicity in human epithelial carcinoma cell lines (RPMI medium) at a concentration of 10 µg/mL [87,88]. This behavior of IONPs can be explained by the charge on the surface of IONPs which plays an important role in the intracellular uptake. However, these findings cannot be correlated with every case. Citrate-IONPs were found to be more genotoxic compared to bare and TEOS-IONPs at the dose of 200 µg/mL in murine fibroblast cell line (L-929 cells). Authors explained this citrate induced damage due to transport of citrate-IONPs through nuclear membrane which generated hydroxyl radicals resulting into DNA damage [89]. Attempts were also made to study the biocompatibility and cytotoxicity of positively and negatively charged IONPs in a mouse brain-derived microvessel endothelial cell line (bEnd.3). Authors observed no significant toxicity by positively charged IONPs in bEnd.3 cells whereas 25% reduction in cell viability was found in the astrocytes (dose: 224 µg/mL, 24 h incubation) in the presence of magnetic field. On the other hand, negatively charged IONPs showed significant toxicity in astrocytes and neurons at higher doses (>100 μg/mL). Interestingly, the cellular accumulation of negatively charged IONPs was less than that of positively charged IONPs in astrocytes. But, negatively charged IONPs showed more toxicity in IONPs indicating the major role of functional groups in toxicity [90].

Different types of cytotoxicity assays, cells/tissues, and culture media can also provide variations in *in vitro* toxicity results. Selection of cell lines is influenced by the major exposure routes of IONPs. For example, the lung is one of the main exposure routes, so it would be more logical to use the murine alveolar macrophage cells to obtain reliable *in vitro* cytotoxic results. IONPs have been tested with a variety of cell lines such as human epidermal keratinocytes, human lung epithelial cell lines, BRL3A rat liver cells, and Cos-7 monkey fibroblasts [91]. However, response of IONPs was found to be altered by the type of cell lines. Amine-modified IONPs induced 25% reduction in cell viability of astrocytes from a mouse brain derived endothelial cell line (DMEM media) at a concentration of 224 μg/mL [62] whereas amine modified MNPs showed little reduction in human dermal fibroblasts and human fibrosarcoma cells at the same concentration (DMEM media) [62]. Dose dependent effects of IONPs have been verified with different cell types. IONPs often induce cytotoxicity at concentrations greater than 300 μg/mL and prolonged exposure time [84,92,93]. The studies conducted with murine macrophage (DMEM media) [92] and human lung alveolar epithelial cells (DMEM media) [94] found that the cell death associated with increasing concentration is due to the generation of ROS mediated oxidative stress. Typically, the clinical dose of IONPs is between 0.015–0.075 Fe/kg [95] which is very low compared to the doses used in *in vitro* studies. Clearly, the difference between the *in vitro* and clinical dose of IONPs is significant and can be explained by the motive of studying the tolerance and biocompatibility of IONPs at higher doses. Knowing the toxicity of IONPs at higher doses could provide guidelines for further biodistribution studies and also shed light on the possible mechanism of toxicity generation.

Physical damage by IONPs can also cause toxicity by inducing oxidative stress in cells. Incubation with IONPs affects the cell surface roughness which could also change the shape and alter the response by cellular cytoskeleton [96]. Morphological changes can be monitored by electron microscopic techniques such as scanning electron microscopy (SEM)/cryo-transmission electron microscopy (TEM) or atomic force microscopy (AFM) [97] *In vitro* assays such as lactate dehydrogenase (LDH) measures leakage of LDH from the cell membrane upon incubation with IONPs and indicative of damage to cell membrane Some of the other assays that can provide information about cell membrane integrity are annexin V which labels phosphatidylserine, propidium iodide staining, and neutral red staining [98].

Several studies appeared in the literature discussing the effect of the type of culture medium on the cytotoxic results of IONPs. The 3-dimensional culture medium has been increasingly preferred by scientists due its ability to mimic a natural cell environment, which provide a larger surface area for interaction with drugs or NPs. Also, it has been found that cell-cell interaction in 3D culture medium is similar to behavior of cells *in vivo* [99]. In contrast, traditional 2D medium culture cells on the solid surface such as plastic or glass fail to provide 3D information [100]. Toxicity studies of IONPs in PAEC grown in 3D and 2D culture media have shown increased cell death in the case of 3D culture media when compared to 2D media. The disparity can be due to improved interaction of IONPs with cells in 3D media [83]. On the contrary, Luo *et al*. found the significantly lower toxicity of bismuth NPs, quantum dots, and IONPs in microtissues grown in 3D media compared to the tissues grown in 2D media. The 3D microtissue culture forms a protective layer that may inhibit the uptake of NPs [101], and the cells inside the microtissue structure can repair quicker than in 2D culture. These experiments were conducted by using a high throughput toxicity 3D microtissue array approach. In this approach, cells were grown in 96 well agarose coated microplates, allowed to aggregate and form microtissues, and then treated with NPs [102].

Recently, multiparametric toxicity evaluations by high content screening (HCS) are being implemented to perform *in vitro* toxicity investigations of IONPs [103]. Multiparametric evaluations can allow assessment of the toxicity of IONPs in terms of multiple parameters such as cell viability, proliferations, cellular physiology, and also provide real time data to monitor cellular processes, which conventional techniques fail to do. IONPs can be tested with multiple cell lines in multiple doses using the HCS approach in both qualitative as well as quantitative settings. Recently, multiparametric toxicity analysis of aminodextran (ADNH), aminopropylsilane (ASi), dimercaptosuccininc acid (DMSA/OD10) coated IONPs with MCF7, BT474, and MCF10A cells was reported. This approach monitored cell viability, cell membrane permeability, and lysosomal pH of cells exposed to different doses of IONPs simultaneously [102]. We have assembled recently published *in vitro* toxicity studies of IONPs focusing on physicochemical parameters in Table 1. However, it is impossible to mention every single *in vitro* report; selections were primarily made on the basis of type of coating agents used for IONPs. Apart from the conflicting results generated by *in vitro* testing, it still provide a primary direction in the long run of toxicity studies and hints at the complexity involved in IONPs mediate toxicity. Impact of several physicochemical properties of IONPs on *in vitro* toxicity assays increases the probability in variations of results. This process has become more efficient with the help of HCS, which have provided relief by generating larger set of data in a small amount of time. But, a proper characterization of IONPs must be performed to explain the variations in *in vitro* studies.

**Table 1:** Brief overview of recent *in vitro* cytotoxic studies of iron oxide nanoparticles (IONPs), organized with emphasis on physicochemical parameters of IONPs. Majority of IONPs discussed in these studies were spherical in shape.

Coating Agent	Types of IONPs	Diameter (nm)	Type of Cells	Dose	Incubation Time	Types of Assay	Brief Results	Ref.
Silica	Bare IONPs	10 ± 3	Human dermal fibroblasts (HDFs) and human fibrosarcoma (HT-1080) in DMEM media	200–1000 μg/mL	24 h	CCK-8 and LDH	APTMS-TEOS-Fe_3_O_4_ showed more cytotoxicity in terms of metabolic activity compared to other MNPs in HDFs. All MNPs induced LDH leakage in HDFs and HT-1080 cells.	[62]
	TEOS-IONPs	100–150						
	APTMS-TEOS-IONPs	100–150						
	Bare IONPs	10–50	Peripheral blood lymphocytes in RPMI media	1–100 μg/mL	2 and 24 h	Annexin V-FITC apoptosis detection	No significant difference between treated and untreated lymphocytes for 2 and 24 h.	[104]
	VTES-TEOS-IONPs	10–50						
	APTES/VTES-TEOS-IONPs	10–50						
	Bare IONPs	150–200	L929 fibroblasts in DMEM media	15–1000 mg/L	24–72 h	MTT	Silica coating reduced cell toxicity. Sulfhydryl modification improved cell-compatibility and haemocompatibility.	[105]
	TEOS-IONPs							
	DMSA-TEOS-IONPs							
	TEOS-IONPs	15–20	MCF-7 and HeLa cells in DMEM media	0–200 μg/mL	24 h	MTT	MCF-7 and HeLa cells showed good biocompatibility at various concentrations.	[106]
PEG	PEG-IONPs	~30	Hela cells and C6 cells in DMEM media	0.01–1 mg/mL	12 h	MTT	Cell viability was not affected at the concentration of 1 mg/mL.	[107]
	PEG-IONPs	10–15	NIH/3T3 in DMEM	1.5 to 192 μM	24 and 48 h	MTT	PEG-IONPs showed good compatibility, 86% (24 h) and 67% (48 h) at 192 μM.	[108]
	Bare IONPs	10–13	Macrophages (mice) in RPMI media	100 μg/mL	1 h	MTT	No significant changes in viability after 1 h by all IONPs. Bare IONPs produced highest ROS compared to PEG and COOH-PEG-IONPs.	[109]
	PEG- IONPs	100						
	COOH-PEG-IONPs	100						
	PEG-550-IONPs	8–11	Bovine vascular smooth muscle cells (VSMCs) in DMEM media	100–1000 ppm	5–24 h	LIVE/DEAD viability/Cytotoxicity Kit	Dose dependent cytotoxic response was found. PEG-2K showed higher cell viability compared to PEG-10K at 100 ppm.	[110]
	PEG-2K-IONPs							
	PEG-5K-IONPs							
	PEG-10K-IONPs							
	PEPABC: IONPs	36 ± 5	Mouse brain endothelial cell line (bEnd.3) in DMEM media	0–10 mg/mL	30 h	Resazurin dye assay	No cell death reported after 30 h exposure at 10 mg/mL.	[111]
Dextran	Dextran-IONPs	200–250	Head and neck squamous cell carcinoma: tonsilla (UT-SCC-60A) and the metastasis (UT-SCC-60B) in DMEM media	0.2–1.8 mM	0–120 h	MTT, Annexin-V-apoptosis detection assay	MTT: Decreased cell toxicity of dextran-IONPs compared to Resovist^®^ Annexin-V-apoptosis: no changes in cell viability when cells were treated at the concentration of 1.8 mM.	[112]
	Dextran-IONPs	100	Mouse melanoma cells (B16) and Chinese hamster lung; fibroblast cells (V79) in DMEM media	0–400 μg/mL	24 h	MTT	Slight changes in the cell viability were noticed as compared to control.	[113]
	Dextran-IONPs	9.12 ± 1.46	L929 fibroblast cells	50–1000 μg/mL	24 h	MTT	Significant reduction in cell viability at 1 mg/mL. Cells were 90% viable at 0.75 mg/mL.	[114]
	DEAE-dextran-IONPs	27–50	Murine mesenchymal stem/stromal cell (MSC) in DMEM media	50 μg/mL	3 h	CCK-8	No significant changes I the cell viability were noticed.	[115]
	Bare Fe_2_O_3_	7	Human bone marrow mesenchymal stromal cells (hBMSCs) hBMSCs-1: age 12 years; hBMSCs-2: age 54 years in α-modified eagle media (α MEM)	15.4 g of iron/mL	72 h	WST-1	The study compared physicochemical properties of bare Fe_2_O_3_ and nanoparticles coated with different coating agents. hBMSCs-1: significant reduction in cell viability by PLL-Fe_2_O_3_and mannose-Fe_2_O_3_ NPs; hBMSCs-2: reduction in cell viability by all IONPs, mostly by uncoated-Fe_2_O_3_ and PLL-Fe_2_O_3_ NPs.	[116]
	Endorem^®^ (Fe_3_O_4_ coated with dextran)	5.5						
PLL	PLL-Fe_2_O_3_	5.5						
PLL-dextran	PLL-Endorem^®^	5.6						
PDMAAm	PDMAAm-Fe_2_O_3_	7.5						
Mannose	Mannose-Fe_2_O_3_	7						
Mono-meric citrate layer	IONPs-*R*_1_	6.5–7.5	Murine primary brain cells (primary microglia, primary hippocampal neurons, and neuron–glia co-cultures) in DMEM media	0.5, 1.5 or 3.0 mM	6–24 h	PI staining	Extended incubation and dose dependent cell death was observed by all IONPs except Ferumoxytol. Ferumoxytol surprisingly increased the number of viable cells. IONPs-*R*_1_, *R*_2_ and Ferucarbotran were quickly ingested by microglial cells compared to Ferumoxytol.	[117]
	IONPs-*R*_2_	7.5–8.7						
Carboxy-dextran	Ferucarbotran (Resovist^®^)	60						
Carboxymethyl-dextran	Ferumoxytol (Feraheme^®^)	30						
Chitosan	Bare IONPs	50-100	Human L-O_2_ hepatocytes in RPMI media	1.25–20 μg/mL	24 h	MTT	Bare IONPs showed more cytotoxicity compared to FAPLCS-IONPs in L-O_2_ hepatocytes.	[118]
	FAPLCS-IONPs	136.60 ± 3.90						
	Bare IONPs	18	Primary human osteoblast cells (SV40) in DMEM media	20–300 μg/mL	48 h	CCK-8	Decreased viability found when cells were treated with bare IONPs at 100 and 300 μg/mL.	[119]
	CS-IONPs	35						
	CS-IONPs	2–8	Cervical carcinoma cell lines (HeLa and SiHa)	0–1000 μg/mL	24 h	XTT	Bare and CS-IONPs showed reduction in cell viability by 5% and 2% respectively. SiHa cells showed 8% reduction in cell viability at 1000 μg/mL.	[120]
Carbon	Fe@C/C	5–140	Human (HTB140), murine (B16-F10) melanoma cells and human dermal fibroblasts (HDF) in DMEM	0.0001–100 μg/mL	24 h	MTT	Decreased cell viability in melanoma cells. Murine melanoma cells were more sensitive to bare IONPs than human cells. Fe@C-COOH and Fe@C-CH_2_CH_2_-COOH showed weaker response to cells, and 80%–100% cells remained viable.	[121]

Abbreviations: TEOS: tetraethyl ortho silicate, APTMS: (3-aminopropyl) trimethoxysilane, PEG: polyethylene glycol, VTES: triethoxyvinylsilane, FITC: fluorescein isothiocyanate, PLL: poly-l-lysine, DMSA: *meso*-2,3-dimeraptosuccinic acid, XTT: (2,3-bis-(2-methoxy-4-nitro-5-sulfophenyl)-2*H*-tetrazolium-5-carboxanilide), PEG-CS-PTH NPs: parathyroid hormone (PTH 1−34) loaded PEGylated chitosan nanoparticles, PEG-(550,2K,5K,10K)-IONPs: IONPs coated with PEGs of varying chain length, FAPLCS: folate-conjugated *N*-palmitoyl chitosan micelles, DEAE-dextran-IONPs: diethylamino ethyl (DEAE)-Dextran coated IONPs, PEPABC: (poly(ethylene glycol)-poly(aspartate) block copolymers), CS-IONPs: chitosan coated IONPs, Fe@C/C: bare carbon encapsulated IONPs, Fe@C-COOH, Fe@C-CH_2_CH_2_-COOH: carboxylic acid modified IONPs.

### 3.2. In Vivo Toxicity Evaluation of IONPs

Despite the rapid, inexpensive and reproducible nature of *in vitro* studies, little correlation has been made between *in vitro* and *in vivo* studies [122]. This is partly due to the inability of *in vitro* studies to mimic the complex environment and homeostasis mechanism maintained by clearance organs such as the kidney and liver. However, to proceed with registration of a drug with FDA for human clinical trials, *in vivo* studies explaining biocompatibility, biodistribution, and bioclearance in animal models must be completed. *In vivo* studies are considered a critical step to study the pharmacokinetic parameters such as absorption, distribution, metabolism, and excretion of IONPs. Irrespective of the expenses and ethical issues involved with *in vivo* studies, they remain to be an integral part of research studies aiming towards gaining a better understanding of IONPs in the body.

Similar to *in vitro* studies, various physicochemical factors such as surface chemistry, size, shape, and charge of IONPs play an important role in animal studies [91]. To reduce immediate uptake of IONPs by the liver and spleen, researchers have concentrated on improving the circulation of IONPs in the bloodstream by developing monodispersed nanocrystals or incorporating biocompatible coating agents. Encapsulation of IONPs in a biocompatible layer has shown increased residence time and reduced accumulation in the liver and spleen [123,124]. Conjugation of *meso*-2,3-dimercaptosuccinic acid (DMSA) to PEG-IONPs doubled the residence time upon intravenous administration [125]. *In vivo* studies conducted with positively charged IONPs have shown improved penetration of placenta in CD-1 mice than negatively charged COOH modified IONPs [126]. Size-dependent toxicokinetics studies demonstrated favored uptake for small IONPS (10 nm) by the liver, rapid clearance by kidneys in mice, and uptake of large IONPs (40 nm) by the spleen [36].

Different exposure routes can affect primary interactions and intracellular entry of IONPs in cells. Organs, which are enriched with reticuloendothelial systems (liver, spleen, and lungs), take up the majority of IONPs introduced by most of the administration routes. IONPs administered by inhalation route and intravenous route accumulated in the liver, spleen, brain, testis, and lung, whereas IONPs administered by an intravenous route in mice were accumulated in the kidney, spleen, and brain [123,127]. Intraperitoneal injections of IONPs in mice also crossed the blood brain barrier (BBB) without affecting its function. Authors suspected the involvement of circumventricular organs of the brain in the uptake of IONPs [128]. Circumventricular organs of the brain lack BBB, and can be an alternative route for anticancer drugs, peptides, and hormones [129].

Different types of animal models have also displayed variable toxicity profiles of IONPs. Changes in the levels of aspartate transaminase (AST) and alanine transaminase (ALT), which is indicative of liver function, were reported in different types of animals upon intravenous administration of IONPs. Increased levels of AST and ALT were found after 1 month of intravenous injection in mice. Moreover, hematology studies conducted on mice showed slight increase in the neutrophils content after one day which returned to normal one month post injection [123]. Another *in vivo* study with pluronic coated IONPs showed normal levels of AST and ALT after 3 days of intravenous injection in rats [124]. The type of animal models used (mice and rat) and preparation methods for IONPs can be responsible for this disparity.

Data obtained from physicochemical characterization and *in vitro*/*in vivo* toxicity studies of IONPs can be considered as an important factor for successful transition to clinical setting. However, comprehensive understanding of the quantitative distribution of IONPs in the various organs of the body to determine the iron clearance holds a key to successfully extrapolate nanomedicines to clinical setting. Thus, it is required to equally emphasize the *in vitro*/*in vivo* quantitative analysis of IONPs in different organs to assign pharmacokinetic profile to IONPs nanomedicines.

## 4. Quantification of IONPs

**Figure 2:**
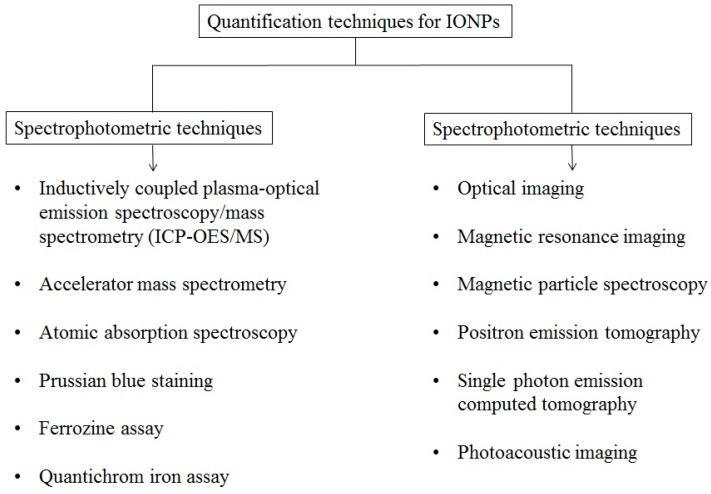
Major spectrophotometric and imaging based quantification techniques for IONPs.

The significant amount of work poured into developing nanodelivery systems have raised the need to study the pharmacokinetic parameters of nanomaterials, which can be used to improve the efficacy of the system with minimal side effects. Accurate quantification of nanodelivery systems containing IONPs is a critical step that provides information about biodegradation and bioclearance patterns of IONPs. Quantitative analysis of intracellular IONPs in various cellular compartments such as mitochondria, lysosomes, and nuclei may provide directions towards their biological impact, and assist in designing potential nanocarriers. Iron quantification techniques can be broadly classified on the basis of spectrophotometric and imaging based measurements (Figure 2). Inductively coupled plasma-optical emission spectroscopy (ICP-OES) or inductively coupled plasma-mass spectrometry (ICP-MS), fluorescence, and UV-Vis measurements [112,113] such as Prussian blue staining, ferrozine assay, and Quantichrom iron assay™ are a few of the commonly used spectrophotometric techniques for quantification of IONPs. Noninvasive, imaging based techniques such as fluorescence, magnetic resonance imaging (MRI), positron emission tomography (PET) and single photon emission computed tomography (SPECT) and optical imaging allows *in vivo*/*in vitro* quantification of IONPs with better sensitivity and reproducibility [114,115].

### 4.1. Spectrophotometric Quantification of IONPs

Inductively coupled plasma-optical emission spectroscopy (ICP-OES) or inductively coupled plasma-mass spectrometry (ICP-MS) are considered as a “gold standard” technique to quantify IONPs due to their high detection limit [130]. ICP-OES can quantify iron without any additional labeling at a concentration of 0.1 mg/L (0.1 ppm), whereas ICP-MS can detect iron at 0.1 to 100 ppt. Several studies have shown the potential of ICP-OES and ICP-MS in biodistribution studies [117,118,119,120]. In addition to ICP-AES and ICP-MS, atomic absorption spectrometry (AAS) was also utilized to quantify the IONPs and to study the biodistribution of IONPs [131,132]. Size-dependent uptake of IONPs was studied in the liver, spleen, kidneys, heart, lungs, brain, intestine, stomach, and uterus in female Kunming mice using AAS. Small IONPs (10 nm) were easily taken up by the liver, whereas the spleen showed the highest uptake of large IONPs (40 nm) [36]. Since ICP-AES, ICP-MS and AAS quantify IONPs by detecting Fe^2+^ or Fe^3+^, digestion of IONPs is required to release Fe^2+^ and Fe^3+^ prior to analysis, thereby making these technique strictly useful for *in vitro* or *ex vivo* purpose. Most importantly, these techniques fail to differentiate between the iron from IONPs and endogenous iron, which is in the form of ferrin, ferritin, and hemoglobin. Additionally, the expensive nature and unavailability of ICP-OES/AES or ICP-MS in biomaterials laboratories limits the access to this quantification approach. Precaution should also be taken in preparation of proper calibration standards, which should mimic the environment in tissues and body fluids.

Biodistribution of IONPs can also be tracked using sensitive isotope-based labeling accelerator mass spectrometry (AMS). AMS is commonly used for radiocarbon dating, which accelerates the ions to extremely high kinetic energies followed by separation of isobars according to their respective mass to charge ratios [133]. The same approach can be applied to studying the long term *in vivo* biodistribution pattern of ^14^C NPs [134]. AMS was also utilized to study the pharmacokinetics and biodistribution of ^14^C labeled IONPs administered by the inhalation route. The AMS successfully detected ^14^C labeled IONPs in the lungs, liver, and spleen with a detection limit of 1 ng/mg [103].

**Figure 3:**
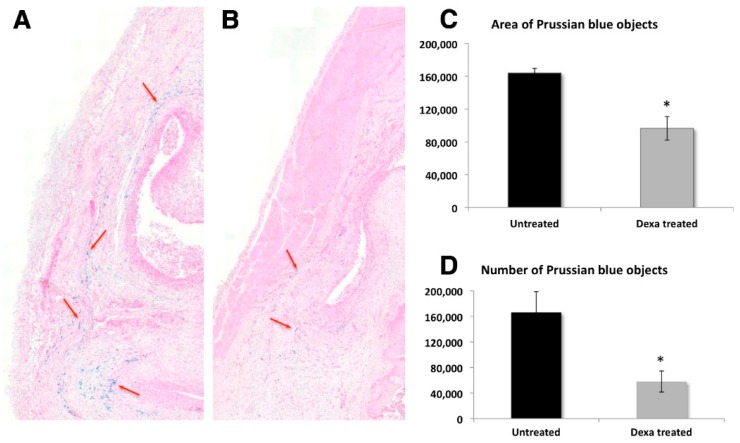
Distribution and quantification of Prussian blue stained superparamagnetic iron oxide nanoparticles (SPIONs) on day 13 post-antigen-induced arthritis (AIA) induction. Photomicrographs of Prussian-blue-stained sections showing an example of the distribution of SPIONs (red arrows) in the synovium of untreated animal (**A**) versus a Dexa-treated animal (**B**) on day 13 post-AIA induction at 1.5 times magnification. Quantification of the area (**C**) and number (**D**) of Prussian-blue-stained SPIONs on day 13 post-AIA induction. Photomicrographs of Prussian-blue-stained sections were scanned, and the images were analyzed for the area (**C**) and the number (**D**) of SPIONs using Tissue Studio® software. Four sections were quantified and averaged per animal. Data points are mean ± standard error of the mean and *n* = 5 per group. * *p* = 0.005 (**A**) and 0.016 (**B**) compared to the untreated control group. Reprinted with permission from Gramoun *et al.* [141] Copyright 2014 Biomed Central Ltd.

In order to perform rapid and affordable quantification of IONPs, many laboratories rely on colorimetric techniques that involve the use of easily available UV-Vis spectrophotometer or fluorescence spectrophotometer. The majority of colorimetric methods modify the iron ions with the reagents that can absorb light at certain wavelengths. Colorimetric ferrozine assay has been acknowledged as a reliable method for measuring intracellular iron uptake. The simplicity and affordable nature of this technique has gained popularity for *in vitro* quantification of IONPs [36,126,127,128,129]. The 3-(2-pyridyl)-5,6-bis(4-phenylsulfonic acid)-1,2,4-trizine (Ferrozine) forms a complex with Fe^2+^ ions to yield a magenta colored solution which absorbs light at 562 nm. The complex is stable at a pH range of 4–10, and has a detection limit in the range of 2 to 10 ppm. Ferrozine also forms colored complexes with Cu, Co, Ca, Mg, Pb, Ag, Mo, Al, Ni, Zn, As, Mn, Cr, oxalate(>500 mg/L), cyanide, and nitrite [135,136]. Initially, Ferrozine was shown to form a complex with the reduced iron form only. However, recent work by Im *et al*. noted the interference of Fe^3+^ in ferrozine measurements in the absence of light, which resulted in an increase in the absorbance upon increased incubation [137]. Additionally, due to photosensitive nature of ferrozine-Fe^2+^, precaution should be taken while performing the measurements [138]. Another simple and high throughput colorimetric technique, Quantichrom iron assay, was used to determine the *in vitro* iron uptake in cells. Upon reduction of Fe^3+^ to Fe^2+^, the chromegen reacts with Fe^2+^ and forms a blue colored complex, which can be measured at 590 nm [139,140]. Another chromophore, Bathophenathroline, forms a complex with Fe^2+^ that can be measured at 525 nm. Ascorbate treatment is used for the reduction of Fe^3+^ to Fe^2+^ prior to its reaction with bathophenathroline [135,136,137,138]. *In vitro* quantification of IONPs can also be performed by using Prussian blue staining, which involves reaction between potassium ferrocyanide and Fe^3+^ to form a blue product called Prussian blue. Prussian blue labeled Fe^3+^ ions can be measured at 680 nm using a microscope as well as a spectrophotometer [130]. In order to monitor the glucocorticoid treatment for rheumatoid arthritis in rats, Prussian blue stained IONPs were utilized to provide qualitative and quantitative information for the distribution pattern of IONPs (Figure 3). In this work, Prussian blue staining was used to verify the MRI tracking procedure in rats and was found to correlate with MRI results [141]. In another work, Prussian blue staining was utilized to evaluate the effect of the charge of IONPs on crossing the placenta in pregnant CD-1 mice. Positively charged IONPs were found to be accumulated in the fetal liver, whereas negatively charged IONPs caused negligible toxic effects [126]. Conflicting results with histological analysis of IONPs by Prussian blue staining were noticed by Scharlach *et al*. [142]. High levels of iron in aortic roots and chondrocyte-like cells of male apoE (negative) mice were found, which were in disagreement with results reported by Langheinrich *et al*. Authors attributed this disparity to differences in protocol and use of thicker slices [143].

### 4.2. Imaging Based Quantification of IONPs

Optical imaging techniques such as fluorescence have been preferred by scientists due to its sensitivity and ease of use over radiolabeling techniques. To quantify the IONPs by fluorometric methods, the IONPS are encapsulated with coating agents containing fluorophores [128] conjugated to quantum dots [144], or containing the fluorophore embedded in their core. Highly sensitive and nondestructive fluorometric quantification of IONPs can be performed by using a confocal laser microscope [145,146] or fluorescent activated cell sorting (flow cytometry) [147,148]. A variety of fluorophores were reported that can be used in conjunction with IONPs. However, the ability of iron oxide (exogenous and endogenous) to quench the fluorescence signal remains a major hurdle in fluorescence-based quantification studies. The conjugation/coupling chemistry to incorporate fluorophore with IONPs can also affect the stability of IONPs-fluorophore assemblies. The photosensitivity of fluorophore, physicochemical parameters of IONPs, and interference from the heterogeneous medium (cell interior) can also affect the fluorescence measurements [149].

Near infrared (NIR) fluorescent dye labeled IONPs can be quantified by fluorescence reflectance imaging (FRI). However, due to weak tissue penetrating ability of 2D FRI, a 3D fluorescence molecular tomography (FMT) was introduced and reported to have spatial resolution of ~500 μm with ~10–20 mm tissue depth [150]. FMT can be combined with diffuse optical tomography (DOT) to clear heterogeneous optical tissue distribution [151]. Recently, IONPs decorated with amino terminal fragment (ATF)-NIR830 dyes adduct were utilized to conduct biodistrubution studies in mouse model with 4T1 cells. The IONPs were accurately quantified using DOT corrected FMT and provided better resolution than FMT without DOT [152].

Ability of superparamagnetic IONPS to shorten transverse relaxation (*T*_2_ and *T*_2_*) and show negative contrast have made them a favorable choice to use as an MRI contrast agent [153,154]. A linear relationship exists between the decay of MRI signal (1/*T*_2_ and 1/*T*_2_*) and the quantity of iron at low concentrations [152,153,154]. The linear correlation has been further utilized to quantify and track IONPs in the brain, heart, central nervous system, and tumors in tissues [154,155,156,157,158]. The *R*_2_*-based quantification has shown some pitfalls such as overestimation of relaxation rates due to air tissue interface [155,156], and complication due to free or non-compartmentalized iron oxide [157]. Moreover, MRI quantification depends on the localized iron concentration, which means that the total concentration of iron oxide decreases due to distribution in continually proliferating cells, thereby affecting the MRI signal [158].

To avoid the interference of endogenous iron in *R*_2_* quantification, AC magnetic susceptibility measurements (MSM) have been preferred for *in vivo* quantification of IONPs [159,160]. Upon applying the AC magnetic field, changes in AC magnetization, in terms of in phase and out phase susceptibility, can be recorded. Due to the different out phase susceptibility of endogenous and exogenous iron (IONPs) at different temperatures, differentiation can be easily performed on the basis of AC susceptibility measurements. MSM measurements are easy, rapid, require no pre-modification of samples, and have a detection limit of 1–2 ppm. MSM measurements were found to be more accurate in the presence of endogenous iron for quantification and biodistribution studies compared to ICP-OES and Prussian blue staining [161]. Another technique, which successfully differentiated the quantity of endogenous and exogenous iron (IONPs), utilized the different *M*(H) curves of endogenous iron and IONPs. The saturation magnetization (*M*_s_) can be easily calculated by using the Langevin function. The *M*_s_ calculations were used for *in vivo* quantification of dextran-coated IONPs in the liver and lungs of Balb-c mice, and the values were found to be 0.25 and 2.4 μg, respectively [162]. Electron spin resonance (ESR) can also distinguish between endogenous and exogenous iron with great sensitivity (30 nmol Fe/kg), and demonstrated *in vivo* and *ex vivo* quantification of IONPs [163]. In a comparative analysis of ESR and ICP-OES to quantify IONPs in a glioma bearing rat model, ICP-OES was unable to detect IONPs in the tumor, brain, and kidney compared to ESR. The insensitivity of ICP-OES was attributed to the presence of background endogenous iron [164].

MRI quantification has proven to be effective at low concentration since higher IONPs concentration can cause MRI signal loss and image distortions. Pitfalls of *R*_2_* based measurements of MRI signal at higher concentrations have directed researchers towards *T*_1_ quantitative mapping sequences, such as ultra-short echo time (UTE) and sweep imaging with Fourier transformation (SWIFT). The UTE and SWIFT can reduce the time interval between excitation and acquisition to microseconds, thereby minimizing the *T*_2_* signal loss. The sweep imaging with Fourier transformation using variable flip angles (VFA-SWIFT) allows the ability to perform *T*_1_ measurements by varying the flip angle close to Ernst angle, the peak signal intensity for a given *T*_1_. This ability of VFA-SWIFT can allow the *T*_1_ quantification of IONPs with higher concentration (1–7 mM) [165,166].

In another technique called magnetic particle spectroscopy (MPS) or magnetization response spectroscopy, frequency and large amplitude is applied to IONPs and the non-linear magnetization response of IONPs in the presence of an oscillating magnetic field is monitored [167]. The MPS was recently utilized to quantify IONPs in Hela and Jurkat cells with the detection limits of 5 ng for Resovist, 50 ng for Ferraheme, and 100 ng for carboxydextran coated IONPs [168]. MPS measurements utilized small sample volumes (5 µL), allowed rapid measurements, and a high throughput screening (200 samples per hour). Aggregations of nanomaterials have shown to affect the MPS measurements due its effect on Brownian motion of NPs [169,170,171].

“Hot spot” techniques such as positron emission tomography (PET) and single photon emission computed tomography (SPECT) can be combined with MRI or computed tomography (CT) to provide qualitative and quantitative information about anatomical and morphological changes. PET utilizes positron emitting radionuclides for visualization and quantification with detection limit in picomolar range. Radionuclides can be introduced in the IONPs during synthesis steps either in the coating layer [172,173] or in the core of IONPs [174,175]. Core labeled ^59^Fe monodisperse IONPs were synthesized and tested in both *in vitro* and *in vivo* systems. The ^59^Fe labeled IONPs were found to be stable in various organic solvents and biological media (fetal bovine serum), and no morphological changes were observed. The ^59^Fe IONPs were injected in mice via tail vein injection with minimum dose (to avoid radio toxicity) to study size and time dependent half-life of polymer-coated IONPs. The ^59^Fe IONPS were found to be stable in biological media (fetal calf serum), and no morphological alterations were observed. The same work also observed an entirely different organ uptake pattern by ^14^C labeled IONPs raised a possibility of separation of the ^14^C label in biological media [172]. The similar results were reported by Wang *et al*. while studying the *in vivo* integrity of IONPs labeled with ^111^In. Some degree of dissociation of ^111^In from IONPs was also reported [169]. The ^59^Fe and ^111^In labeled IONPs showed a similar biodistribution pattern; however, ^51^Cr labeled IONPs showed excretion in feces rather than in urine which can be attributed to a different path of metabolism of ^51^Cr [170]. Widespread use of radiolabeled quantification techniques is hindered by requirement of skilled personnel, special instrumentation, and health hazards of radionuclide exposure.

**Figure 4:**
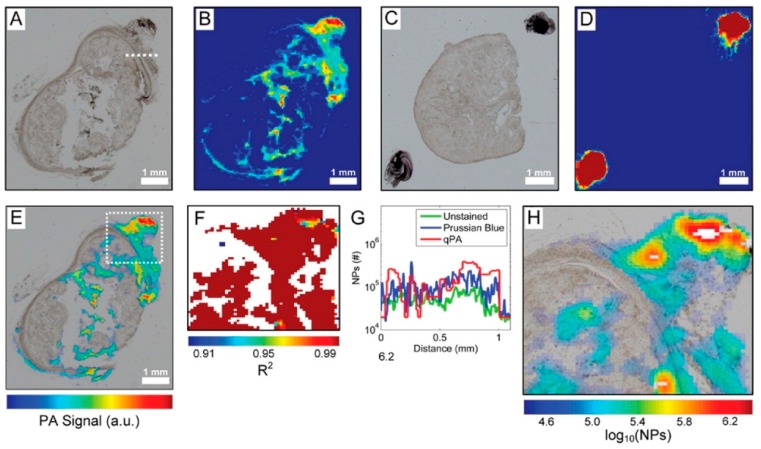
Bright-field microscopy (**A**,**C**) and photoacoustic (PA) (**B**,**D**) images of unstained tumor slices with (**A**,**B**) and without (**C**,**D**) NPs. An overlay of the optical and PA image of the tumor with NPs from (**A**) and (**B**) are shown in (**E**). *R*^2^ map of the area in the white box in (**E**) is shown in (**F**); Quantitative PA image using *R*^2^ > 0.97 with unquantifiable areas in white is shown in (**H**); (**G**) Quantitative comparison of the unstained and Prussian blue stained bright-field images and the qPA images. The values for the graphs were generated from a line shown in (**A**). Reprinted with permission from Cook *et al*. [171] Copyright 2015 American Chemical Society.

Quantitative photo acoustic imaging was demonstrated to accurately quantify IONPs in both *in vitro* and *ex vivo* settings [171]. When an optically absorbing subject interacts with a pulse of light, the light energy is converted into heat energy, which upon absorption by the surrounding medium, creates acoustic waves. Thus, photoacoustics is a product of absorption coefficient and the local fluence. In addition to endogenous absorbers such as hemoglobin, IONPs can be used as an optically absorbed medium [176]. Using a custom made photoacoustic microscope, IONPs were accurately quantified in thin slices of xenograft epithelial tumor with a detection limit of 2 × 10^4^ NPs per spot. In Figure 4, the difference in the photoacoustic signal of the tumor slices with (Figure 4A,B) and without NPs (Figure 4C,D) can clearly be seen. This highly sensitive *ex vivo* approach can also preserve spatial distribution, which renders it effective for *in vivo* applications [171].

Non-invasive and sensitive nature of imaging based techniques certainly has an edge over spectrophotometric based quantitative techniques. No single imaging technique can single handedly provide good depth, resolution and sensitivity, which explains the emergence of multimodality imaging agents.

## 5. Conclusions and Perspective

An enormous amount of cytotoxic studies appeared in the literature to improve the current understanding of toxicity of IONPs. However, inconsistencies keep growing with an increasing number of research studies. A striking disparity found between the shape, size, and coating dependent toxicity of IONPs directs the focus towards a meticulous physicochemical characterization of IONPs. Discrepancies between *in vitro* and *in vivo* cytotoxicity results can also be rooted in the physicochemical properties of IONPs, and more importantly affected by the behavior of IONPs in biological media. The interaction of IONPs with cells and tissues can alter the physicochemical properties, mainly the aggregation of IONPs. Thus, knowing the altered surface chemistry of IONPs in the *in vivo* setting can help to develop a link between the surface chemistry of IONPs and its relevant induced toxicity.

Sterilization of IONPs is one of the ignored factors that contribute towards the toxicity of IONPs. The importance of sterilization of pharmaceuticals is highlighted and prioritized in various pharmacopoeias. However, the majority of studies lack or fail to report the sterilization data prior to *in vitro* or *in vivo* use of metal oxide NPs. IONPs should be tested for microbial contaminations (bacteria, yeast, and mold) to assure the safety of IONPs, and to eliminate a parameter, which could lead to ambiguous cytotoxic results. The majority of IONPs can be considered sterile due to harsh synthesis procedures; however, contamination can be introduced in various downstream processes such as surface functionalization. Sterilization can be performed by filtration, irradiation, and autoclaving or treating with various organic solvents containing disinfectant properties [177].

Passivating the hydrophobic IONPs with a hydrophilic layer is a common practice during synthesis of biomaterial agents, which improves the solubility and stability in aqueous and biological solvents. However, the integrity of a hydrophilic layer needs to be maintained at various pHs in the cellular environment. Various biological environments can remove this hydrophilic coating, and the bare IONPs can be directly exposed to cells. Considering the altered and higher toxicity of bare IONPs, it is important to monitor the stability of biocompatible IONPs in *in vivo* experiments, and the toxicity studies should be conducted in an environment, which mimics the cellular environment.

The majority of IONPs induce toxicity in cells via ROS production, which varies according to physicochemical properties of IONPs. Considering the role of physicochemical properties of IONPs in ROS production, stricter rules to determine the oxidative properties of IONPs should be followed. The high reactivity and short half-life of ROS are major obstacles faced while studying the mechanism of IONPs.

Advanced, sensitive MRI and PET/CT scanners have changed the face of *in vivo* quantification of IONPs, and have helped to gain a better understanding of the *in vivo* distribution and clearance patterns of IONPs. However, many biomaterials laboratories do not have access to expensive MRI instruments and prefer spectrophotometric techniques for quantification. Choosing an appropriate quantification technique from a large pool of options depends on a variety of circumstances. Considering the pros and cons of every quantification technique, a combination of multiple techniques may be necessary for the absolute quantification of IONPs in *in vivo* setting. In addition to quantification of intracellular IONPs, surface ligand quantification [178] should also be taken into consideration since it influences the interaction of IONPs with proteins and other biological components.

Some encouraging research studies have been published about the pulmonary and dermal toxicity of IONPs. When inhaled, engineered airborne NPs can cause severe harm such as asthma, chronic obstructive pulmonary disease, pulmonary inflammation, and lung cancer [179]. From the respiratory tract, they can translocate to secondary organs such as the lymphatic or circulatory system [180]. Development and deployment of high-throughput methods for sensing and diagnosing NPs exposure and their mechanism of interaction, is thus an important unmet need across all branches of the biological sciences and nanotechnology. Failure to meet this need exposes all biological systems to the risk of irreversible, long-term NP exposure and the corresponding health consequences it shares with other poorly metabolized byproducts (e.g., synthetic estrogens from birth control medications). To avoid these effects, a set of strict and rational guidelines aiming to minimize the direct exposure of IONPs should be followed by the growing community of scientist dealing with IONPS.

Current progress in the applications of IONPs promises upcoming excitements, with a hope of successful transformation of under trial and pre-clinical nanomedicines to a successful pharmaceutical agent. Significant barrier between the research based nanomedicine and commercialization is the vast structural complexity of nanomaterials that differentiates them from marketed drugs in terms of lacking a clear “definition” and is of utmost importance in studying their *in vivo* bio-distribution and clearance pattern. Unfortunately, the toxicity and biodistribution studies have failed to keep up with the rapid pace of synthesis of novel nanomaterials and require similar attention. As presented in this review, we hope to provide a clear understanding of the physicochemical factors that need to be monitored during *in vitro* and *in vivo* toxicity studies of IONPs. Additionally, this document will also assist researchers in selection of the combination of proper quantification techniques to monitor the fate of IONPS in the body.
